# Meeting Report

**DOI:** 10.1155/S1463924697000084

**Published:** 1997


Journal of Automatic Chemistry, Vol. 19, No. 2 (March-April 1997) pp. 51-54

### Meeting Report

### *ROBOTICS AND AUTOMATION IN THE PHARMACEUTICAL LABORATORY*

### *Introduction*

*The use of automation and robotic modules in a range of pharmaceutical and other fine chemical analysis laboratories was the subject of a meeting organized by the Automatic Methods Group and the Joint Pharmaceutical Analysis Group, held at the Royal Pharmaceutical Society's headquarters in London, on 7 December 1995. Current scope and opportunities for the future were considered, speakers from diverse fields discussed a range of successful applications. An equipment exhibition was also organized, enabling participants to see recent developments in the automation of dissolution testing and the analytical processing of metered-dose inhalers.*

### *Abstracts of papers presented*

### State of the art in analytical robotics

**Kevin Warwick**
*University of Reading*

In the opening presentation, Professor Kevin Warwick (Department of Cybernetics, University of Reading) updated the traditional concepts of robots (from those of either humanoid machines or standard production line arms) by describing intelligent, decision-making systems which feature a number of different information sensors to control movement and to select actions to be carried out. Modern robotic systems are now capable of learning by experience and are able to pass on this information to other robots.

Various new developments were described, incorporating a number of new technologies, perhaps the most impressive of which concerned the process control of bank-note production. In order to prevent forgery, the colour combinations used in the printing of banknotes are as complicated as possible and, although some tolerances are allowable, there are limits. Accordingly, a suitable robotic system for this application needs an appropriate feedback mechanism which allows a correct quality judgement to be made.

Similarly, cigarette manufacture relies on largely uninterrupted operation and it is extremely important to be able to predict or to schedule stoppages. This multi-variable situation is difficult for humans to control, however, but predictability can be obtained by using an appropriate computer-based algorithm.

Professor Warwick discussed the applicability of artificial neutral networks in attempting to mimic some of the operations carried out by the human brain. As such networks are good at modelling non-linear systems, they can be used directly to control robot manipulators or to assess the influence of a large number of process variables. The use of such technology, typically in a manufacturing or laboratory environment, will result in improved process control and operational performance.

### Microbiological assays

**Professor Ken Coleman**
*SmithKline Beecham*

Some automated applications of microbiological assays were described by Ken Coleman (SmithKline Beecham, Brockham Park) in which the minimum inhibitory concentration of an antibiotic is established using a screening experiment involving, typically, as many as 40 organisms.

The first application featured the automation of the Hamilton AT + incorporating an X-Y arm capable of holding up to 12 syringes, and a liquid level detection system. This facilitated preparation of microlitre plates more rapidly than was achievable manually (typically, a reduction from 12 minutes to four minutes).

The overall process of plate preparation (remembering that 40 organisms are routinely involved in the screening procedure) and plate reading was able to be completed in minutes, rather than several hours, with the automated system—which also incorporated in orca robot to stack plates for the Hamilton AT+, and a barcode reader for sample identification.

A second approach involved the use of a Zymate robotic system with image analysis facility, multi-point inoculator and an agar dispenser. This system also proved suitable for the kind of high-volume screening applications common to many microbiological laboratories, with the additional advantage that correct plate alignment could be assured routinely.

### Progress and speculation

**Ken Leiper**
*Glaxo Laboratories*

'History repeats itself—because we don't learn from it!' was the opening provided by Dr Ken Leiper (Glaxo Wellcome, Barnard Castle) in his provocative presentation which reviewed progress in, and considered the future of, the application and automation of analytical measurement.

The development of automated measurement has shown considerable progress over the last three or four decades,

0142-0453/97 $12·00 © 1997 Taylor & Francis Ltd from the dedicated auto-analysers of the 1950s to the vastly more sophisticated intelligent solutions of the present day where chemometric data handling techniques and robotic control are common. Moreover, analytical science itself is now generally recognized as having a major part to play in the way in which measurement techniques develop.

The changing environment in which most businesses operate is forcing a reappraisal of the way in which automated measurements are being carried out. Corporate objectives and regulatory requirements are becoming increasingly more demanding, with products needing to be introduced more rapidly, with increased efficiency, more consistent quality and generally with reduced head count.

Current measurement philosophy seems to fall some way short of the mark when these new business objectives are considered. Typically, laboratory-based methodology is developed in research and will need to be transferred into development, into manufacture—and perhaps even beyond, into a stability or contract group. Moreover, such methodology tends to be product-focused, with significant sample preparation, and gives little or no information about process performance. Demonstration of compliance, from the regulatory perspective, also seems to rely on highly bureaucratic procedures involving extensive checking and documentation.

Thus, the role of the analytical scientist is to provide an effective measurement service which helps to fulfil corporate objectives. This necessitates a measurement philosophy which establishes the relationship between 'need', 'measurement' and 'technology'.

Methodology is required which features little or no sample preparation, which may be easily transferred—particularly to the production area, if necessary—and which is self-calibrating and self-validating. Two examples were provided of such an approach; an automated antibiotic assay system which allowed measurement of several product quality parameters within a cycle time of only five minutes with overall software control to provide and maintain system integrity; and the use of rear infra-red spectroscopy to provide chemical and physical information directly on the sample matrix (for example, during powder blending or tabletting studies).

Such approaches, where emphasis is placed on process measurement rather than purely on the product, provide advantages in terms of reduced costs, improved process reliability, ease of method transfer and increased assurance of process and product compliance. Changes are required, however, in terms of recognition of business needs, improved liaison between suppliers and academia and the development of an improved skills base.

### On-line near infra-red

**Malcolm Crook**
*Process Analysis and Automation*

Dr Malcolm Crook (Process Analysis and Automation, Farnborough) initially discussed the benefits which are achievable from the improved control provided by process analysis. These include the production of materials of consistent quality, increased throughput and efficiency, reduction in waste and time—as well as a demonstrable improvement in regulatory/compliance aspects.

In-line process analysis is particularly beneficial in that rapid (real time) results may be obtained, no sampling steps are required and operations generally minimize the exposure of staff to hazardous materials. The suitability of near infra-red spectroscopy for in-line analysis was emphasised in view of the speed and applicability of the technique, its relative insensitivity towards process debris, colour of sample, etc. and the fact that it utilizes sensible path lengths (unlike infra-red spectroscopy, for example).

Incorporation of fibre optic probes further enhances the capabilities of in-line process analysis using near infra-red spectroscopy. Both reflectance and transmission probes can be used, while fibre optic bundles up to 1 or 2 km in length are also available. Such bundles can also be multiplexed, so that fibres may be made to point to different channels by simple perturbation of the electromagnetic field in which they are held.

One consequence of the use of near infra-red spectroscopy is the need to apply some chemometric data analysis to the measurements obtained. Near infra-red bands are generally broad, poorly resolved and often difficult to assign. The application, therefore, of chemometric techniques such as 'partial least squares', for example, can prove extremely useful in correlating spectral information with chemical composition, physical aspects and general product quality characterization.

### Automation in water analysis (a laboratory for the 21st century)

**Robin Andrew**
*North West Water*

North West Water's automated water analysis laboratory was described by Dr Robin Andrew (North West Water Ltd, Laboratory Services). The company provides water and waste-water services over an area of 14,000 $km^2$ and has about seven million customers. The Laboratory Services group provides an analytical service to the company on a range of materials (raw water, waste-waters, trade effluents, sludges, soils etc.) and with a range of analyses (organic and inorganic species, bacteria, parasites, etc.)—all within a very heavily regulated environment.

Typically, the Laboratory Services group handles over 250,000 samples per year and, with the benefits of automation, has reduced the number of its laboratories from 30 to 1 and its staff from 260 to 110 since 1989.

The single major laboratory was designed with the objectives of improved quality of data, faster reporting of analytical results and lower operating costs. These aims have been achieved by establishing segregated areas, each dedicated to a particular type of analysis, through which samples are transported via a standard conveyor belt system. Different robotic units are used in each analytical area, typically 'industrial' type robots or 'gantry' style systems. Barcode readers are used for sample identification. Within the laboratory, some analytical instrumentation has deliberately not been fully integrated, in order to avoid becoming too committed to particular systems.

Overall control of the laboratory is provided by a LIMS, with manual intervention in the event that any of the automated checking and validation procedures should fail.

A number of lessons were learned during the design and implementation of the automated water analysis laboratory: clear objectives are essential, these laboratories need a lot of space, flexibility is vital, system integration requires careful planning and, crucially, *staff involvement* is the most important part of the exercise.

### Automation in pesticide residue analysis

**Keith Parsley**
*CSL Food Science Labortory*

Mr Keith Parsley (Central Science Laboratory, Norwich) outlined the way in which automation has been introduced into the Pesticides Group of CSL (a MAFF Executive Agency) for the analysis of pesticide residues in foodstuffs.

The laboratory undertakes a variety of analyses (drug residues, toxicants, environmental contaminants, pesticide residues, etc.), most of which is funded by MAFF and which is, therefore, subject to significant financial constraint. Thus, any moves towards automation have had to be aimed at increasing throughput, but without incurring any large capital expenditure.

Among other activities, the Pesticides Group provides surveillance data on the major constituents of the diet of the UK population as part of a continuous monitoring programme. Thus, a variety of commodities are examined and a large range (well over 100) of pesticides are involved. This has resulted in the development and application of eleven significantly different methods of analysis (incorporating different techniques) to allow this range of pesticides to be monitored.

In order to automate these activities, emphasis has been placed on increasing efficiency, with reduced costs, increased sample throughput and greater staff productivity. Examination of the various stages involved in the pesticide analysis procedure revealed that improvements were possible in the sample clean-up step, where fats or plant colourants are separated from the pesticide residues using gel permeation chromatography. Incorporation of a Gilson 232 sample handling system has improved this aspect significantly, allowing up to 60 samples to be run overnight.

In addition, a sample concentration step (involving an expensive and time-consuming nitrogen evaporation process) has been modified to incorporate a Jouan centrifugal evaporator which can take up to 10 samples, significantly improving the productivity of the operation.

Improvements in other parts of the analytical procedures (for example greater use of auto-injectors and the introduction of a chromatographic data handling system) have resulted in an overall increase in productivity of the laboratory. Automated approaches based on existing technology have been used, and have allowed staff time to be used more effectively.

### Automated applications to bioanalysis

**Gordon Plummer**
*Zeneca Pharmaceuticals*

A review of the application of an automated solution involving a Zymate robotic system was provided by Dr Gordon Plummer (Zeneca Pharmaceuticals). Analysis of samples arising from drug kinetic studies needs to be automated in view of reduced drug development times, increased regulatory expectations and greater economic constraints.

The sample preparation step of such analyses is the rate-determining factor so, to address this problem, Zeneca introduced a Zymate system into the laboratory in 1984. They now have eight such systems, capable of performing a wide variety of analytical tasks, although most have been dedicated to specific applications.

In particular, the robotic systems are engaged in liquid-liquid and solid-phase extraction's, allowing reductions in analysis time from ten minutes originally to less than three minutes now. As such, this has demonstrated the benefits of keeping up-to-date with developments and of up-grading the systems as new technology becomes available.

Extensive review of over several years has shown comparability of robot performance using the same modules, with good correlation between data generated automatically and manually. More extensive computer validation is required these days, however.

In terms of cost benefit, the robotic system was found to pay for itself in less than one and a half years when used for complex analyses and in less than three years for simpler applications, when used continuously for three to four days per week. Dedicated resources are needed, however, if further applications are to be developed and good technical and engineering back-up is also required.

### The self-service spectroscopy laboratory: a blueprint for the future?

**Don Clark**
*Pfizer Central Research, Sandwich*

The principles of 'open access' analytical chemistry were given by Mr Don Clark (Pfizer Central Research,

Sandwich) in a lively concluding presentation. Pfizer have used automation to help overcome the problem of increasing turnaround times in their Physical Sciences Laboratory, allowing the customers to collect and interpret their own data using spectroscopic instruments.

JMR was the first technique to be automated and has progressed from the use of a Nicolet QA 300 system to systems linked to Zymate's robot. This has improved capacity dramatically, freeing up the analysts' time to do more innovative tasks.

The development of an 'open access' mass spectrometer proved slightly more difficult in that commercial instrument manufacturers had not considered such a system at that time. Pfizer were able to collaborate extensively with VG, however, to design a suitable system which eventually fulfilled their needs—and provided the instrument manufacturer with a commercial product.

Polarimetry is used extensively in Pfizer's laboratory and this technique has also now been automated successfully, based around a Perkin Elmer 341 system with custom-made turntable from Peerless, to allow 'open access'. Some 'smart' software developments have also been included for fault/error detection (for example, the detection of air bubbles in the system).

The approach which has been adopted within the Physical Sciences Laboratory has been very well-received by the customers and allows more data, of improved quality, to be generated with less resource. Liaison and collaboration with instrument manufacturers is important in terms of helping to realize the true benefits of an automated approach.

### *Conclusion*

Evidently there is no single way in which robotics or laboratory automation should be used to improve productivity or data quality—a variety of approaches are possible and significant levels of success may be achieved.

One important conclusion which may be reached, however, is that experiences gained within one field of application frequently translate into other areas. Thus, the analytical scientist, charged with the task of introducing the benefits of automation into the laboratory, would be well-advised to consider the successes gained by colleagues working in different sectors. This, and the importance of concentrating efforts on analytical measurements providing information about the process, rather than the product, were the key messages emanating from this well-attended and thought-provoking meeting.

